# Emotional and Behavioral Trajectories of 2 to 9 Years Old Children Born to Opioid-Dependent Mothers

**DOI:** 10.1007/s10802-020-00766-w

**Published:** 2021-01-12

**Authors:** Julia Jaekel, Hyun M. Kim, Samantha J. Lee, Ashlyn Schwartz, Jacqueline M. T. Henderson, Lianne J. Woodward

**Affiliations:** 1 Department of Child & Family Studies, University of Tennessee Knoxville, Knoxville, USA; 2 Department of Psychology, University of Tennessee Knoxville, Knoxville, USA; 3 Department of Psychology, University of Warwick, Coventry, UK; 4 School of Health Sciences, University of Canterbury, Christchurch, New Zealand; 5 School of Psychology, Speech and Hearing, University of Canterbury, Christchurch, New Zealand; 6 Department of Public Health, University of Tennessee Knoxville, Knoxville, USA

**Keywords:** Opioids, Neonatal abstinence/opioid withdrawal syndrome (NAS/NOWS), Developmental trajectories, Biopsychosocial risk

## Abstract

Maternal opioid use in pregnancy has increased dramatically. Knowledge about children’s longer-term emotional and behavioral development after prenatal opioid exposure is scarce. A regional sample of 89 opioid-exposed and 104 non-exposed comparison children were studied prospectively at ages 2, 4.5, and 9 years using the Strengths and Difficulties Questionnaire (SDQ) completed by primary caregivers. Across all childhood assessments, opioid-exposed children obtained significantly higher total difficulties scores than non-exposed comparison children. Growth curve modeling revealed that, relative to their same age peers, opioid-exposed children’s emotional and behavioral difficulties significantly worsened over time. Moreover, fixed effects estimates showed that total difficulties trajectories were poorer for children subject to higher prenatal risk (Est = 1.78, 95% CI = [0.46, 3.09]) who were born to mothers with high levels of social adversity (1.11 [0.51, 1.71]), and were then raised in families characterized by high levels of psychosocial risk (1.94 [0.90, 2.98]) and unstable caregiving (1.91 [0.33, 3.48]). A complex set of pre- and postnatal processes contribute to opioid-exposed children’s emotional and behavioral development. Efforts to mitigate the long-term consequences of opioid use in pregnancy need to consider both children’s and their caregivers’ biopsychosocial risks.

Over the last 20 years, the use and abuse of licit and illicit opioids has skyrocketed globally (Sanlorenzo, Stark, & Patrick, 2018), including among women of reproductive-age (Bateman et al., 2014; CDC, 2017). As a consequence, the number of infants prenatally exposed to opioids has increased dramatically (Bateman et al., 2014; Patrick, Davis, Lehmann, & Cooper, 2015; Winkelman, Villapiano, Kozhimannil, Davis, & Patrick, 2018). Methadone maintenance treatment (MMT) provides advantages over continued illicit drug use and is the most common management approach for opioid-dependent pregnant women (Jones, O'Grady, Malfi, & Tuten, 2008). However, MMT involves chronic fetal opioid exposure placing the infant at risk of neonatal opioid withdrawal syndrome (NOWS) (Conradt, Crowell, & Lester, 2018). Longitudinal studies describing children’s longer-term development after prenatal opioid exposure are scarce (Azuine et al., 2019; Kaltenbach et al., 2018; Lee, Woodward, & Henderson, 2019; Levine & Woodward, 2018).

One of the most critical developmental tasks for children is to successfully regulate their behavior and emotions (Fergusson, Horwood, & Ridder, 2005; Mischel et al., 2011). There is growing suggestion that prenatal opioid-exposure may impact children’s self-regulatory abilities, and has consistently been associated with poorer behavioral control and attention-deficit hyperactivity disorder (ADHD) symptoms (Azuine et al., 2019; Lee, Pritchard, Austin, Henderson, & Woodward, 2020; Levine & Woodward, 2017; Nygaard, Slinning, Moe, & Walhovd, 2016; Ornoy, 2003). In addition, compared with their non-exposed peers, children exposed prenatally to opioids may be at increased risk for emotional difficulties (Nygaard et al., 2016), conduct problems and disruptive behaviors (Azuine et al., 2019; Ornoy, 2003). However, follow-up studies of children exposed prenatally to opioids extending beyond infancy and early childhood are rare and methodologically limited (Fill et al., 2018; Lee et al., 2019; Oei et al., 2017). Specifically, existing studies typically report only cross-sectional outcomes at a single age of assessment. Of the few longitudinal studies that do exist, these are confounded by a reliance on small samples, high sample attrition (Nygaard et al., 2016), lack of tester blinding, no or poor measurement of other drug exposures (Crea, Barth, Guo, & Brooks, 2008; Nygaard et al., 2016), or failure to include a comparison group from the same population studied. This significantly limits our current understanding and interpretation of available data.

Yet, we know from normative population studies that the prevalence as well as the nature and severity of children’s behavioral and emotional problems vary with age (Koumoula, 2012; Lenze & Wetherell, 2011; Sasser, Kalvin, & Bierman, 2016; Spencer, Biederman, & Mick, 2007). For instance, ADHD and conduct problems tend to have their peak onset and highest prevalence in childhood, whereas emotional difficulties generally peak later (Kessler et al., 2004). Anxiety problems, for example, can occur in childhood but have a median age of onset in early adolescence, while rates of depressive symptoms typically rise dramatically during adolescence and peak in young adulthood (Kessler et al., 2004).

At present, it is not known whether prenatally opioid-exposed children follow similar behavioral trajectories as their non-exposed peers. Yet we can only really understand the mechanisms underlying potential problems associated with prenatal opioid-exposure when accounting for normative developmental changes. Methodologically, this can be accomplished by longitudinally administering an established, standardized, and developmentally appropriate instrument such as the Strengths and Difficulties Questionnaire (SDQ) (Goodman, 2001) to assess the development of children exposed to opioids as well as a representative regional comparison group.

A further important issue that is vital to address the longer-term consequences of the growing public health problem of families affected by opioid use is to understand the developmental mechanisms that might place children born to opioid dependent women at increased risk of later problematic developmental trajectories. This is an issue that has, to date, been given scant attention beyond considering a wide range of pre- and postnatal factors as potential confounders. Previous approaches have thus precluded opportunities to separately examine the roles of: a) other adverse prenatal exposures that co-occur with fetal opioid exposure; b) maternal social background factors such as education and marital status that are associated with both opioid use and child outcomes; and, importantly, c) the intervening effects of the quality and stability of the postnatal rearing environment in shaping child outcomes (Azuine et al., 2019; Crea et al., 2008). The latter are especially important since although they may be correlated with the independent variable of interest (prenatal opioid exposure) they occur after pregnancy and thus are more accurately viewed as intervening factors that may mediate or moderate associations between opioid exposure and child outcomes. Furthermore, knowledge about the contributions of the postnatal environment to children’s behavioral trajectories is critical in informing intervention efforts aimed at supporting these and future cohorts of opioid-exposed children and families longer term. Specifically, it is critical to identify variables in children’s environments that can be modified through intervention, in order to facilitate resilience for this at-risk group. Most importantly, almost no studies have examined the complex interplay of prenatal exposures, social background, and postnatal environmental factors that may heighten or mitigate risk for infants exposed to opioids. We propose that a biopsychosocial framework may help incorporate the multimodal and multidisciplinary theoretical aspects mentioned above (Bolton & Gillett, 2019). Conradt, Crowell, and Lester (2018), for instance, have proposed an integrated model of how different factors such as socioeconomic status, maternal psychopathology and nutrition may alter opioid-exposed infants’ development prenatally, and they propose that effects could extend into the postnatal period, for instance via mother-infant interactions (Conradt et al., 2018). In line with this model, Fig. 1 shows a schematic depiction of the hypothetical pathways of interest considered in this study. The potential importance of each of these key processes or risk factors is also reviewed briefly below.

**Fig. 1:**
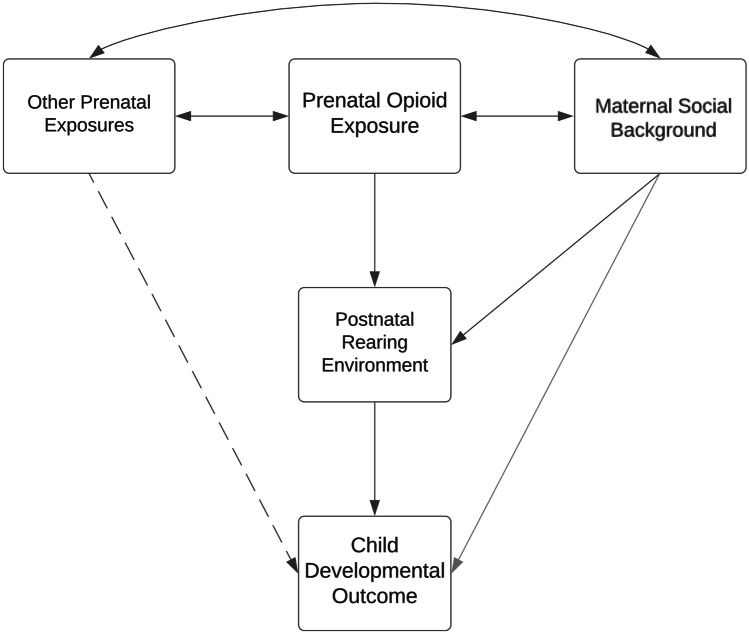
Schematic Model of the Hypothetical Pathways of Interest

With regard to prenatal and neonatal exposures and risks, opioid use in pregnancy has been associated with maternal perinatal depression and psychiatric illness, and smoking during pregnancy, preterm birth, smaller head circumference at birth, and a longer hospital stay (Azuine et al., 2019; Conradt et al., 2019; Davie-Gray, Moor, Spencer, & Woodward, 2013; Kelty & Preen, 2019; Woodward, McPherson, & Volpe, 2018). Postnatally, the quality of infants’ caregiving may be particularly important (Hatzis, Dawe, Harnett, & Barlow, 2017; Konijnenberg, Lund, & Melinder, 2015), but we know remarkably little about whether variations in rearing environments are associated with developmental variability after opioid-exposure.

Children who were prenatally exposed to opioids have been shown to be at increased risk of exposure to psychosocial adversity and poor parental support (Hatzis et al., 2017; Konijnenberg, Sarfi, & Melinder, 2016; Sarfi, Smith, Waal, & Sundet, 2011; Siqveland, Haabrekke, Wentzel-Larsen, & Moe, 2014). However, previous studies have not disentangled the potential effects of prenatal and postnatal factors, but instead treated them collectively as confounders. What’s more, it has rarely been investigated how social background risk indicators may be associated with combined prenatal and postnatal risks when studying children’s long-term development. During pregnancy, there are complex mechanisms at play between maternal factors such as pregnancy nutrition, social disadvantage, and psychiatric risk that are correlated with pregnancy opioid use (Woodward et al., 2018), but their effects on long-term child development have not been investigated among opioid-exposed mother–child dyads. In addition, prenatal (i.e., biological) compared with postnatal environmental factors may affect long-term trajectories via different pathways (Bendersky & Lewis, 1994; Jusiene, Breidokiene, & Pakalniskiene, 2015), and their combined effects may potentially be additive, interactive, or both (Evans, Li, & Whipple, 2013; Li, 2003; Sameroff & MacKenzie, 2003). Sophisticated longitudinal study designs are timely and essential to assess the complex interplay of these biopsychosocial risks on opioid-exposed children’s long-term development.

The aim of this study is to characterize the behavioral and emotional trajectories from age 2 to 9 years of a regionally representative sample of prenatally opioid-exposed children relative to their non-exposed peers. Utilizing a multi-level approach, we address key questions concerning predictive mechanisms of prenatal, social background, and postnatal environmental risk. We formed three hypotheses: Compared with their non-exposed peers, (1) children who were prenatally exposed to opioids will exhibit increased emotional and behavioral difficulties at ages 2, 4.5, and 9 years, (2) their trajectories will remain stable or worsen with age, and (3) prenatal and social risks as well as postnatal rearing conditions will explain individual variations in children’s behavioral trajectories.

## Methods

### Participants

Participants were assessed as part of a prospective longitudinal study of two groups of children born between 2003 and 2008 at Christchurch Women’s Hospital, New Zealand (Davie-Gray et al., 2013; Levine & Woodward, 2017). Mothers were recruited during their third trimester or at birth. Exclusion criteria included very preterm birth (≤ 32 weeks), congenital abnormality, HIV, fetal alcohol syndrome, and non-English speaking.

The index group consisted of infants born to opioid-dependent mothers receiving MMT. Over the recruitment period, 120 mothers were eligible for inclusion in the study. Of these, 99 mothers and 100 infants (including one set of twins) were successfully recruited (84% of all eligible opioid-exposed infants). Retention to age 9 years was 85%. Figure 1 provides an overview of recruitment and retention over the study period. Demographic characteristics are presented in Table 1. A detailed description of recruitment and perinatal medical treatment has been published previously (Davie-Gray et al., 2013; Lee et al., 2020, 2019).

**Table 1:** Descriptive Participant Characteristics, Pregnancy to 18 Months

	Non-exposed	Opioid-exposed	*Mean / % difference*			*p*
**Maternal Characteristics**						
Age (years)	31.64 (5.44)	29.77 (5.26)		-1.87	0.015	
Pregnancy methadone dose (mg)	-	62.63 (35.14)		62.63	< 0.001	
Pregnancy depression (EDPS)	6.83 (4.83)	14.21 (6.13)		7.38	< 0.001	
Pregnancy psychiatric illness (%)	15.89	56.52		36.35	< 0.001	
Pregnancy nutrition score	90.61 (25.31)	56.03 (20.81)		-34.57	< 0.001	
Pregnancy smoking (cigarettes/day)	1.39 (3.88)	13.10 (8.90)		11.71	< 0.001	
% minority ethnicity	17.76	25.00		7.24	0.212	
% younger mother	5.61	3.26		-2.35	0.427	
% single parent	9.35	48.91		39.56	< 0.001	
% no formal education qualification	19.63	80.43		60.80	< 0.001	
Depression at 18 months (EPDS)	5.26 (3.99)	9.79 (6.96)		4.53	< 0.001	
**Infant Characteristics**						
% female	52.34	42.39		-9.95	0.161	
Gestational age (weeks)	39.27 (1.70)	38.82 (1.69)		-0.45	0.063	
Head circumference at birth (z-score)	0.23 (0.86)	-0.24 (0.88)		-0.47	< 0.001	
Total days of morphine	-	63.66 (41.02)		63.66	< 0.001	
Total days in hospital	3.44 (2.29)	16.07 (12.14)		12.62	< 0.001	
Primary caregiver change (%)	4.30	43.00		38.07	< 0.001	
**Composite Scores**						
Prenatal risk (z-score)	0.34 (0.51)	2.18 (1.76)		1.84	< 0.001	
Social risk (sum score)	0.57 (0.93)	1.37 (0.89)		0.79	< 0.001	
Postnatal environment risk (z-score)	-0.26 (0.48)	0.32 (0.63)		0.58	< 0.001	

Data are reported in Mean (SD), if not noted otherwise

**Fig. 2:**
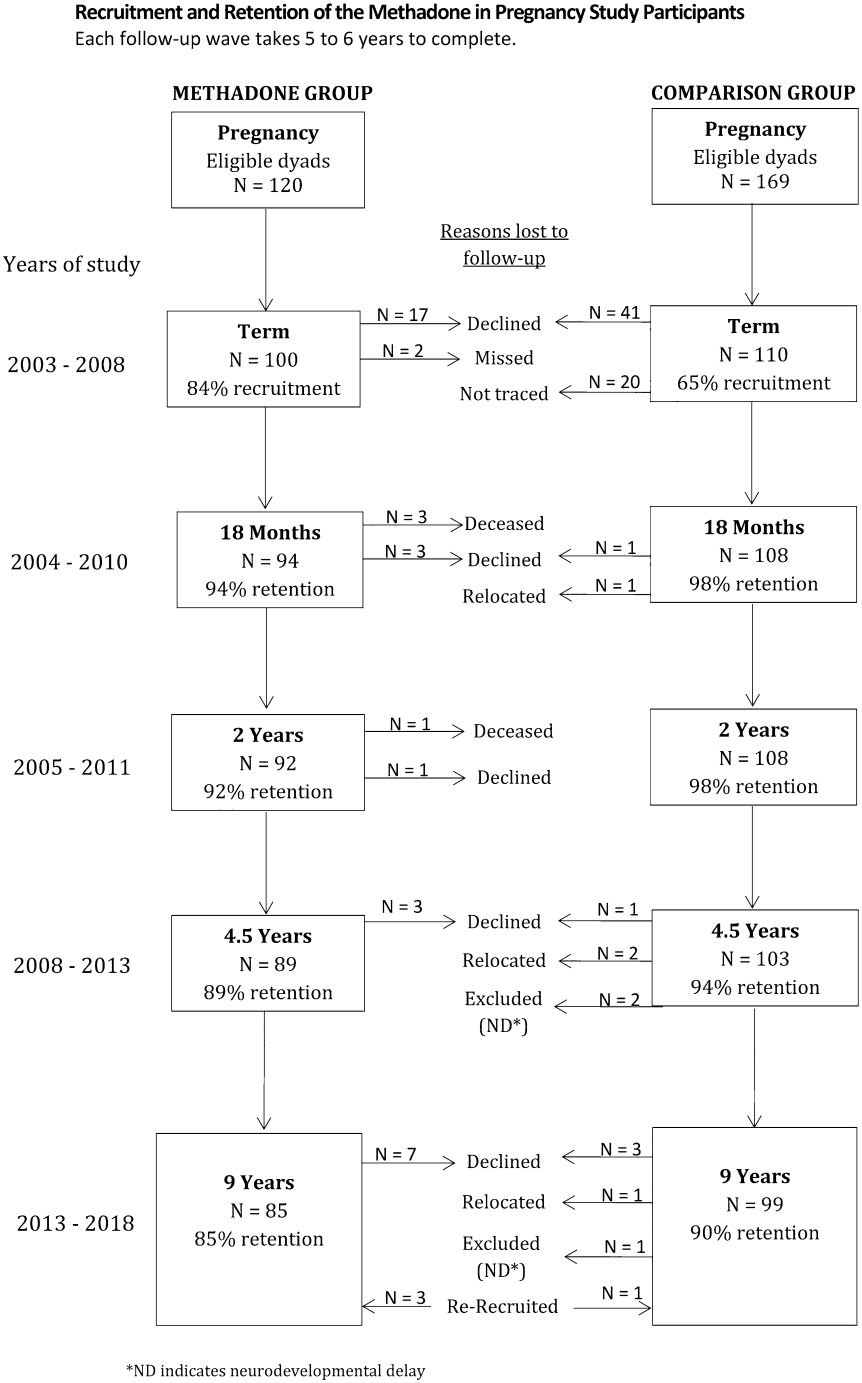
Study Flow Chart

The comparison group consisted of randomly identified non-opioid-exposed infants born at the same hospital over the same recruitment period. A total of 169 pregnant women were approached, with 108 mother and 110 infants (including two sets of twins) successfully recruited. Regional census data showed that the socioeconomic profile of this sample was representative of the families living in the region at the time (Statistics New Zealand, 2006). Retention to age 9 years was 90% (see Fig. 2 and Table 1).

### Procedure

Participants were assessed at five time points: late pregnancy/birth, 18 months, 2, 4.5, and 9 years. The Upper South B Regional Ethics Committee, Canterbury, New Zealand (Ref: URB/07/10/042) approved the study protocol and the study was performed in accordance with the ethical standards as laid down in the 1964 Declaration of Helsinki and its later amendments or comparable ethical standards. Written informed consent was obtained from biological mothers or primary caregivers at each assessment point, with children also providing verbal assent at age 9 years. At each follow-up, participants received a $20 gift card as compensation for their involvement in the study.

### Measures

**Strengths and Difficulties Questionnaire (SDQ)*.*** Primary caregivers completed the SDQ at 2, 4.5 and 9 years during structured parent interviews. The SDQ contains 25-items that measure 5 subscales: emotional symptoms, hyperactivity-inattention, conduct problems, peer problems, and prosocial behavior (Goodman, 1997). Each subscale is scored on a 3-point Likert-type scale (0 = not true, 1 = somewhat true, 2 = certainly true). A total difficulties score is calculated by summing the scores of the four difficulties scales (*Cronbach’s α* = 0.62, 0.70, and 0.75 for the total scores respectively at each age in this study). The SDQ is a widely used screening tool with good test–retest reliability, e.g., *r* = 0.72 for parent-rated total difficulties scores over 4–6 months (Goodman, 2001).

**Maternal Pregnancy/Birth Characteristics*. ***Detailed information about mothers’ mental health, nutrition, and social risks was collected via comprehensive structured interviews administered in the late 3^rd^ trimester or at birth (Lee et al., 2020, 2019), see Table S1 for details.

**Infant Characteristics*.*** Detailed information about perinatal characteristics (e.g., gestational age, head circumference at birth) and infant medical treatment was extracted from hospital records.

We created three risk index scores in order to include a diverse and large set of biopsychosocial risk variables in our analyses, to control for potential multicollinearity between variables, and to increase external validity and reliability.

**Other Prenatal and Perinatal Risk Exposures*. ***A continuously scored index of pre- and neonatal biological risks was created by averaging the following z-standardized variables, thereby weighting their independent contributions equally: infant gestational age, head circumference at birth, 1-min APGAR score, and pregnancy nutrition (all reverse coded), total days in hospital (as an index of postnatal illness severity), smoking during pregnancy, and maternal depression at birth (Edinburgh Postnatal Depression Scale (EPDS), *α* = 0.79) (Cox, Holden, & Sagovsky, 1987). Pregnancy psychiatric illness was included as a binary-coded variable (0 = no, 1 = yes). All variables were equally weighted and averaged, a higher total score indicated higher perinatal risk.

**Maternal Social Risk**. A composite score was created as a summative index of five dichotomous maternal social risk indicators (i.e., single parent, minority ethnicity, no high school degree, low socioeconomic status, mother < 21 years old) (Lee et al., 2020).

**Postnatal Rearing Environment Risk*.*** A continuously scored index of postnatal caregiving quality was created by averaging the following z-standardized variables: the Home Observation for Measurement of the Environment (HOME) total score (*α* = 0.74; reverse coded) (Caldwell & Bradley, 1984; Totsika & Sylva, 2004), parenting stress measured using a 16-item scale assessing caregiver exposure to, for example, financial, housing, social support and relationship stress (α = 0.68), maternal depression at 18 months (EPDS, *α* = 0.81) (Cox, Chapman, Murray, & Jones, 1996; Cox et al., 1987) and 4.5 years (Composite International Diagnostic Interview, *α* = 0.89) (WHO, 1993), corporal punishment at 18 months *(α* = 0.72) (Straus, Hamby, Boney-McCoy, & Sugarman, 1996), and maternal total illicit drug use between birth and 4.5 years (see Table S1). Illicit drug use was continuously assessed at child age 18 months and 4.5 years based on questions from the Composite International Diagnostic Interview (WHO, 1993) about the use/abuse of a range of drugs including heroine, prescription opioids, cannabis, barbiturates, stimulants, hallucinogens inhalants and other illicit prescription drugs (Davie-Gray et al., 2013). The variable was binary coded (0 = no, 1 = yes) for inclusion in the risk score due to limited variance and non-normal distribution.

All variables were equally weighted and averaged, a higher total score indicated higher postnatal rearing environment risk.

**Primary Caregiver Changes*. ***Any transitions or disruptions in children’s primary caregivers were documented and coded as a binary variable (0 = no, 1 = yes). Preliminary analyses showed that primary caregiver changes indicated an independent and specific risk that was not correlated with the more general and normally distributed index score of postnatal rearing environment risk (*Spearman’s rho* = 0.12, *p* = 0.09).

### Statistical Analyses

Descriptive analyses were conducted in SPSS 24 to examine group differences between opioid-exposed and non-exposed comparison children with regard to biological and environmental factors as well as emotional and behavioral difficulties. For the main analyses, a low number of data missing at random was imputed using the stochastic imputation method implemented in “mice 3.8.0” package in R (van Buuren et al., 2020). Imputation was confined to participants with at least two of the three SDQ assessments available, resulting in a longitudinal sample of 89 opioid-exposed and 104 non-exposed children. Correlations between variables included in the prenatal biological and social risk, and postnatal rearing environment scores are presented in Table 2. Trajectories of emotional and behavioral development from 2 to 9 years were assessed using linear mixed effects model analysis in Stata 16. Trajectories of total difficulty scores over time were modeled as a function of age at assessment (years) and group status (opioid-exposed vs. non-exposed), including both fixed and random effects, following an established approach (Mangin, Horwood, & Woodward, 2017). As part of a stepwise process, a quadratic term for age (age*age) was included in Model 2. In Model 3, an interaction term (age*group) was added to test whether trajectories differed between groups. This model was then extended to assess the effects of child sex, prenatal biological and social risk, the postnatal rearing environment, and primary caregiver changes on emotional and behavioral development, including potential additive and interactive effects. Log-likelihood goodness of fit tests were used to compare each growth curve model to the previous one.

**Table 2:** Correlations Between Variables Included in the Neonatal Biological, Social, and Rearing Environment Risk Index Scores

	1	2	3	4	5	6	7	8	9	10	11	12	13	14	15	16	17	18	19	20	21
1. infant gestational age	-	-0.09	-0.07	-0.28	-0.12	-0.22	-0.10	-0.05	0.01	-0.11	-0.08	0.05	0.19	-0.10	-0.08	-0.04	-0.05	-0.08	-0.41	-0.08	-0.17
2. head circumference		-	0.00	-0.19	-0.17	-0.04	0.08	-0.17	0.03	-0.17	-0.20	-0.06	0.08	-0.02	-0.06	-0.17	-0.01	-0.16	-0.29	-0.12	-0.14
3. pregnancy nutrition			-	0.05	-0.08	0.04	-0.19	-0.04	0.03	-0.18	-0.12	0.03	0.02	-0.05	-0.11	-0.03	0.07	-0.06	-0.13	-0.11	-0.06
4. total days in hospital				-	0.44	0.05	-0.04	0.32	-0.03	0.44	0.51	-0.10	-0.33	0.24	0.29	0.32	-0.06	0.32	0.89	0.36	0.38
5. postnatal depression					-	0.17	0.02	0.40	0.06	0.42	0.40	-0.01	-0.29	0.23	0.42	0.34	0.05	0.28	0.55	0.37	0.38
6. pregnancy psychiatr. illness						-	-0.01	0.17	-0.09	0.07	0.00	0.04	-0.11	0.21	0.24	0.06	0.04	0.12	0.21	0.01	0.21
7. pregnancy smoking							-	-0.02	0.19	0.19	0.06	0.14	-0.03	-0.04	-0.08	0.01	0.04	-0.20	0.13	0.11	-0.04
8. single parent								-	0.08	0.47	0.47	0.22	-0.26	0.31	0.30	0.26	0.04	0.31	0.36	0.52	0.38
9. minority ethnicity									-	0.12	0.26	0.20	-0.10	0.07	0.02	0.12	0.03	0.01	-0.04	0.47	0.12
10. no high school degree										-	0.62	0.19	-0.44	0.28	0.33	0.33	-0.05	0.28	0.50	0.58	0.44
11. low SES											-	0.15	-0.40	0.31	0.26	0.37	-0.01	0.37	0.55	0.59	0.47
12. mother < 21 years												-	-0.10	0.04	-0.09	-0.01	0.10	0.02	-0.07	0.25	0.06
13. HOME total score													-	-0.32	-0.27	-0.28	-0.10	-0.31	-0.36	-0.45	-0.61
14. parenting stress														-	0.51	0.27	0.21	0.32	0.27	0.35	0.71
15. mat. depression, 18 m															-	0.43	0.15	0.29	0.33	0.32	0.71
16. mat. depression, 4.5y																-	0.05	0.22	0.36	0.28	0.58
17. corporal punishment																	-	0.15	0.00	0.23	0.47
18. total illicit drug use																		-	0.32	0.33	0.56
19. Prenatal Risk Exposure																			-	0.38	0.43
20. Social Risk Score																				-	0.57
21. Postnatal Rearing Environment Risk																					-

### Results

**Descriptive Sample Characteristics*.*** Indicators of prenatal and social background risk tended to be higher among children in the opioid-exposed group. On average, opioid-exposed infants had lower head circumference z-scores at birth and spent more days in hospital than non-exposed infants. There were however no group differences in infant sex, gestational age, and 1-min APGAR scores. Opioid-using mothers had higher prenatal depression and lower nutrition scores, reported higher average numbers of daily cigarettes smoked during pregnancy, and were subject to higher rates of psychiatric illness. Opioid-exposed infants tended to have been born, on average, to younger mothers than non-exposed children, although rates of early motherhood (< 21 years) did not differ, and they more often had single mothers without formal educational qualifications (Table 1). Rates of minority ethnicity were the same across groups. Accordingly, the prenatal and social risk index scores were higher in the opioid-exposed group. In addition, the postnatal rearing environment risk index score was also higher, and they had more changes in primary caregivers (i.e., care disruptions). For instance, at ages 2, 4.5, and 9 years, respectively, 80%, 69%, and 54% of the opioid-exposed children were in the care of their biological mothers. For the non-exposed group, 100% of children were in the care of their biological mothers at ages 2 and 4.5 years, with 99% in their mothers’ care at age 9 years.

**Emotional and Behavioral Difficulties*. ***Confirming Hypothesis 1, prenatally opioid-exposed children had significantly higher total difficulties scores than their non-exposed peers across all childhood assessments, with the mean difference [95% confidence interval] increasing from 2.06 [0.61, 3.52] at age 2 years to 7.13 [5.30, 8.96] at age 9 years (Table 3, Fig. 2). This was further confirmed by visual inspection of the data in Fig. 2, which suggests that opioid-exposed children’s difficulties appear to increase from ages 2 to 9 years, whereas the scores of children in the non-exposed comparison group tended to remain relatively stable.

**Table 3:** SDQ Subscale and Total Difficulties Scores of Opioid-Exposed Versus Non-Exposed Children at 2, 4.5, and 9 Years

	Non-Exposed			Opioid-Exposed			
**SDQ Score**		**n**	**Mean (SD)**	**n**	**Mean (SD)**	**Mean Difference (95% CI**)	***p***
Emotional Problems	2 y	107	1.36 (1.35)	92	1.45 (1.53)	0.09 (-0.31, 0.49)	0.666
	4.5 y	103	1.27 (1.42)	87	1.90 (1.88)	0.64 (0.16, 1.11)	0.009
	9 y	99	1.74 (1.78)	81	2.98 (2.39)	1.24 (0.61, 1.87)	< 0.001
Conduct Problems	2 y	107	2.28 (1.91)	92	2.71 (2.09)	0.43 (-0.13, 0.99)	0.131
	4.5 y	103	0.92 (1.11)	87	2.47 (1.90)	1.55 (1.09, 2.01)	< 0.001
	9 y	99	1.07 (1.54)	81	2.96 (2.42)	1.89 (1.28, 2.51)	< 0.001
Hyperactivity/Inattention	2 y	107	3.48 (2.42)	92	4.50 (2.47)	1.02 (0.33, 1.70)	0.004
	4.5 y	103	2.44 (2.11)	87	4.29 (2.38)	1.85 (1.21, 2.49)	< 0.001
	9 y	99	2.65 (2.26)	81	5.17 (2.89)	2.53 (1.75, 3.30)	< 0.001
Peer Problems	2 y	107	1.25 (1.50)	92	1.77 (1.79)	0.53 (0.07, 0.99)	0.025
	4.5 y	103	0.93 (1.21)	87	1.59 (1.56)	0.65 (0.25, 1.06)	0.002
	9 y	99	1.23 (1.47)	81	2.63 (2.06)	1.40 (0.86, 1.94)	< 0.001
Total Difficulties	2 y	107	8.37 (4.80)	92	10.43 (5.60)	2.06 (0.61, 3.52)	0.006
	4.5 y	103	5.56 (4.12)	87	10.25 (5.51)	4.69 (3.27, 6.07)	< 0.001
	9 y	99	6.66 (5.00)	81	13.79 (7.00)	7.13 (5.30, 8.96)	< 0.001

**Fig. 3:**
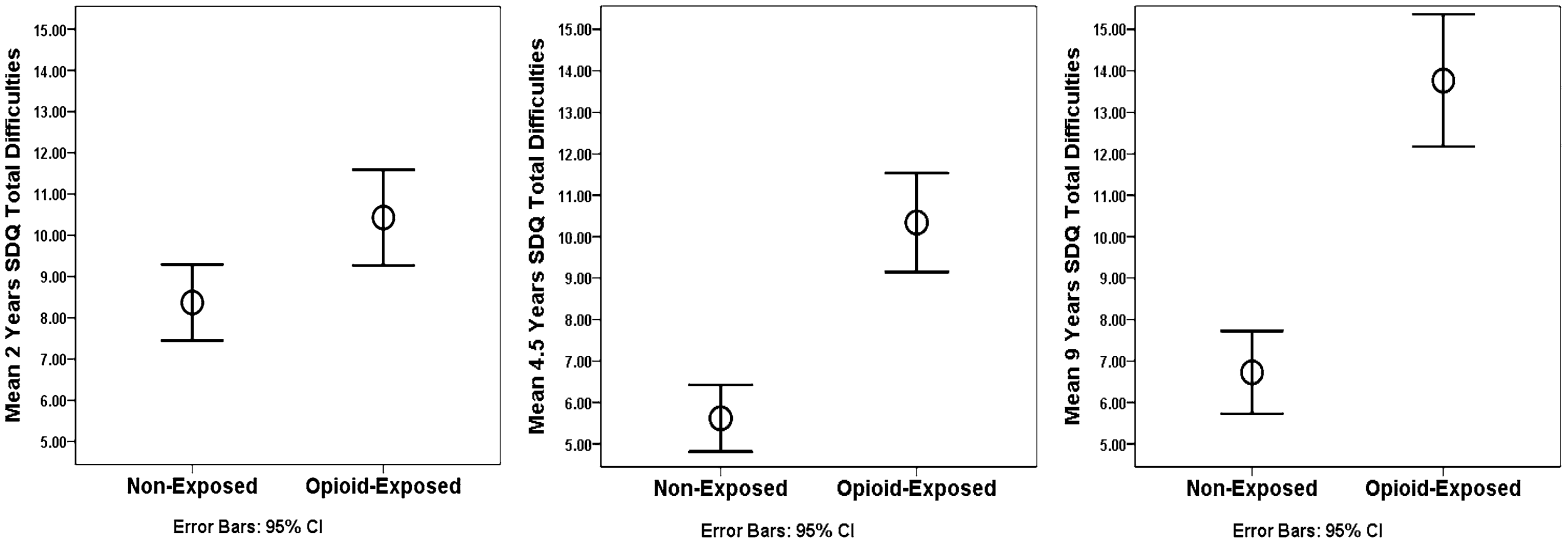
SDQ Total Difficulties Scores of Opioid-Exposed Versus Non-Exposed Children at 2, 4.5, and 9 Years

As part of a sensitivity analysis, we examined the domain-specific nature of opioid-exposed children’s emotional and behavioral difficulties. Table 3 shows the mean scores of the two study groups on each of the SDQ subscales (emotional, conduct, inattention/hyperactivity, and peer problems) as well as the total score by age at assessment (2, 4.5, and 9 years), also see Fig. 3. Across all four domains, compared with their non-exposed same-age peers, there was an increase in opioid-exposed children’s problems from 2 to 9 years. Specifically, opioid-exposed children had higher inattention/hyperactivity (mean difference 1.02 [0.33, 1.70]) and peer problems (0.53 [0.07, 0.99]) than their peers at 2 years, but significant between group differences in emotional and conduct problems were not detected despite the tendency for higher levels to be reported for children in the opioid exposed group. However, by age 4.5 years, opioid-exposed children were found to have higher problem scores across all four SDQ domains, and these group differences further increased at age 9 years.

**Trajectories of Difficulties*.*** Linear mixed effects growth curve models were run to examine the behavioral trajectories of both study groups based on their SDQ total difficulties scores from 2 to 9 years of age, using the stepwise approach outlined above. Table 4 shows detailed results for five models that were subsequently fitted to the data. In Model 1, trajectories of total difficulties over time were modeled as a linear function of age at assessment (years) and group status (opioid-exposed vs. non-exposed). On average, opioid-exposed children had total difficulties scores with coefficient estimates (CE) of 4.23 (95% CIs [3.09, 5.37]) points higher than their non-exposed peers at any given age (*p* < 0.001), while the fixed linear main effect of age at assessment was not significant. Child-specific random variability according to age was significant with an estimated random-slope standard deviation of CE = 2.21 [1.63, 2.98]. The log-likelihood ratio chi-square test supported the use of the mixed effects Model 1 versus a simple linear model (*χ*^*2*^(3) = 71.89, *p* < 0.001).

**Table 4:** Multilevel Growth Curve Model Results of SDQ Total Difficulties Score Trajectories from 2 to 9 Years of Age (*n* = 193)

Total Difficulties	Model 1 Est (SE)	Model 2 Est (SE)	Model 3 Est (SE)	Model 4 Est (SE)	Model 5 Est (SE)
*Fixed effects*					
Age	0.39 (0.25)	-6.57 (1.31)***	-10.08 (1.48)***	-10.08 (1.48)***	-10.08 (1.48)***
Age^2^	-	1.74 (0.32)***	1.74 (0.32) ***	1.74 (0.32)***	1.74 (0.32)***
Group (opioid-exposed)	4.23 (0.58)***	4.23 (0.58)***	-0.13 (1.05)	-3.91 (1.15)**	-4.61 (1.17)***
Interaction (group*age)	-	-	2.40 (0.48) ***	2.40 (0.48)***	2.40 (0.48)***
Sex	-	-	-	-0.52 (0.52)	-0.45 (0.51)
Prenatal Risk	-	-	-	2.03 (0.67)**	1.78 (0.67)**
Social Risk	-	-	-	1.18 (0.31)***	1.11 (0.31)***
Postnatal Environmt. Risk	-	-	-	1.56 (0.52)**	1.94 (0.53)***
Primary Caregiver Change	-	-	-	-	1.91 (0.80)*
constant	1.94 (1.01)	7.74 (1.47)***	14.12 (1.94)***	20.41 (2.20)***	19.09 (2.25)***
*Random effects*					
SD (Age)	2.21 (0.34)*	2.42 (0.30)*	2.11 (0.31)*	2.11 (0.31)*	2.11 (0.31)*
SD (constant)	4.68 (0.75)*	5.16 (0.66)*	4.67 (0.67)*	3.99 (0.72)*	3.88 (0.73)*
correlation (Age,constant)	-0.72 (0.08)*	-0.76 (0.06)*	-0.70 (0.08)*	-0.74 (0.08)*	-0.74 (0.08)*
Log-likelihood	-1,769.61	-1,755.90	-1,744.16	-1,720.39	-1,717.62

for fixed effects *p < 0.05; **p < 0.01; ***p < 0.001; for random effects * marks a 95% confidence interval not including 0

To test for potential nonlinear effects of age on trajectories over time, a quadratic term (age*age) was included in Model 2. As a result, the fixed linear (CE = -6.57 [-9.13, -4.01]) and quadratic effects of age (CE = 1.74 [1.11, 2.37]) were both significant with improved fit over Model 1, indicated by a reduced log-likelihood value (Table 3), and a significant log-likelihood ratio chi-square test (assumption Model 1 nested in Model 2: *χ*^*2*^(1) = 27.42, *p* < 0.001).

In Model 3, an interaction term (group*age) was introduced to test whether behavioral trajectories differed between groups. Again, model fit improved significantly (assumption Model 2 nested in Model 3: *χ*^*2*^(1) = 23.48, *p* < 0.001) with the addition of this new parameter (CE = 2.40 [1.46, 3.34]). The significant group x age interaction in Model 3 implies that opioid-exposed children’s total difficulties trajectories differed from non-exposed children, with total difficulties scores worsening with age, and thus confirming Hypothesis 2.

To test Hypothesis 3, Model 4 then included fixed effects of child sex, prenatal biological and social risk, as well as the postnatal rearing environment composite scores. While child sex did not predict total difficulties trajectories, the previous effects of age and group remained significant and all three risk scores had additionally significant effects. Thus, Hypothesis 3 was confirmed: children with higher prenatal biological risk (CE = 2.03 [0.70, 3.35]), higher social background risk (CE = 1.18 [0.58, 1.79]), and higher postnatal rearing environment risk (CE = 1.56 [0.55, 2.57]) had higher total difficulties scores. Again, model fit improved significantly (assumption Model 3 nested in Model 4: χ2(4) = 47.55, p < 0.001).

In Model 5, a fixed effect of having had any primary caregiver changes on total difficulties trajectories was additionally added (CE = 1.91 [0.33, 3.48]), and this further improved overall model fit (assumption Model 4 nested in Model 5: χ2(1) = 5.54, p < 0.019). Importantly, the group and group by age differences remained significant, even after accounting for related risk factors, which also remained significant.

Finally, we examined possible interactive effects between prenatal biological and social risk, as well as with postnatal rearing environmental influences on children’s overall difficulties trajectories. None of these interaction effects reached statistical significance.

**Fig. 4:**
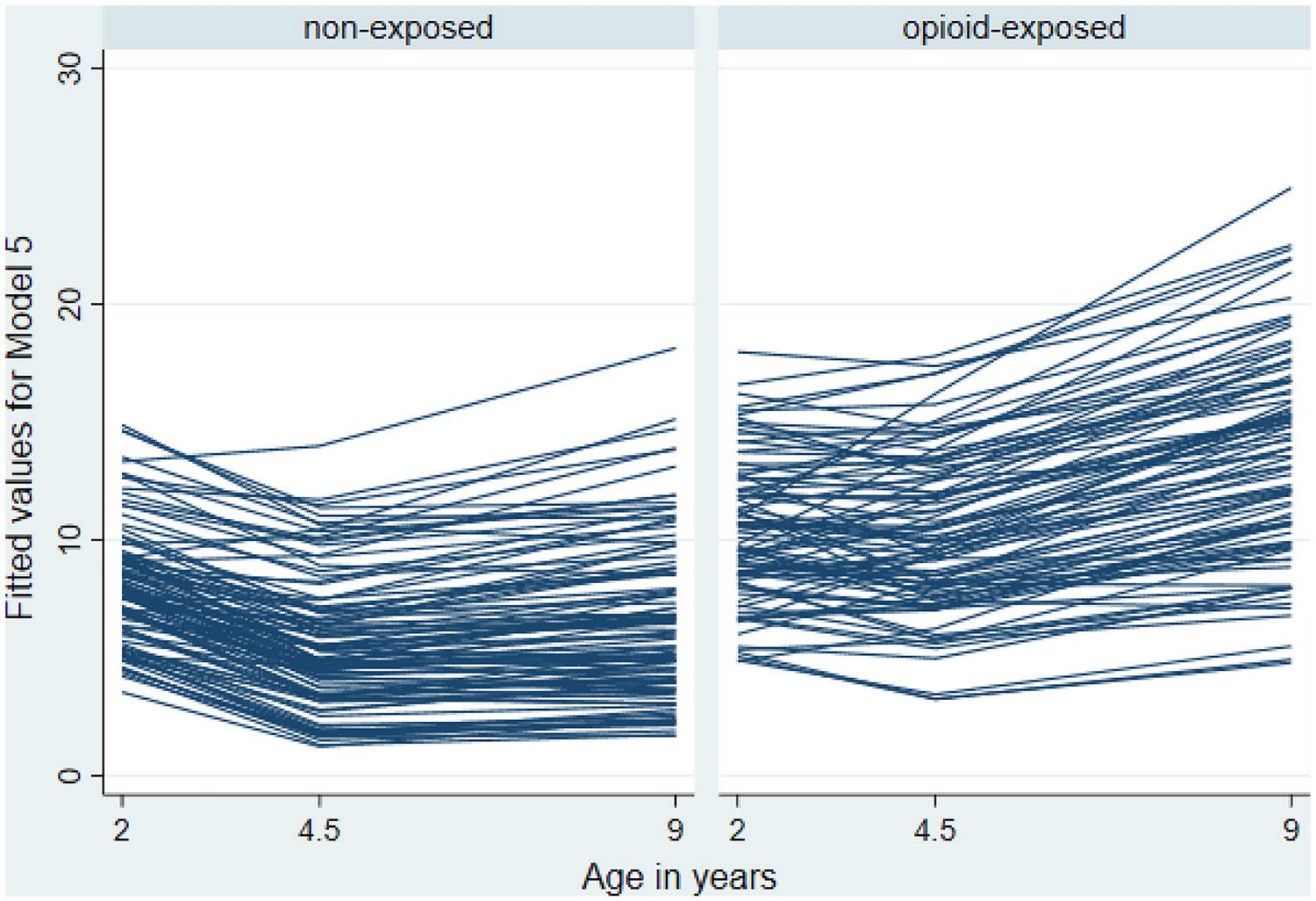
Predicted (Model 5) Total Difficulties Scores Trajectories of Opioid-Exposed Versus Non-Exposed Children from 2 to 9 Years

Thus Model 5, showing independent (i.e., additive) fixed main effects, is considered the final model. Figure 4 shows predicted total difficulties trajectories by group based on Model 5. Overall, opioid-exposed children’s scores were characterized by higher total difficulties and greater inter- and intra-individual variability from baseline (2 years) to 9 years of age.

### Discussion

This is the first prospective analysis of the emotional and behavioral development of an unselected regional sample of opioid-exposed children from birth to 9 years, relative to their same age typically developing peers. Using a hypothesis-driven, multi-level analysis approach, we confirmed all three hypotheses we had formed a-priori. Specifically, compared with their non-exposed peers, (1) children who were prenatally exposed to opioids exhibited significantly higher emotional and behavioral difficulties at 2, 4.5, and 9 years of age. Growth curve modeling further revealed that, (2) relative to their same age peers, opioid-exposed children’s difficulties trajectories worsened over time. Finally, results showed that (3) the index scores of prenatal risk, social risk, and postnatal rearing environment risk we had formed, as well as changes in primary caregivers, independently and additively explained individual variations in children’s behavioral trajectories (see Fig. 4).

In addition to SDQ total difficulties scores, we also examined the domain-specific nature of opioid-exposed children’s emotional and behavioral difficulties in comparison to their same-age peers. An assessment of the existing literature shows that prenatal opioid-exposure is consistently linked with externalizing behavior problems, and in particular behavioral dysregulation and attentional difficulties (Azuine et al., 2019; Levine & Woodward, 2017; Nygaard et al., 2016; Ornoy, 2003; Slinning, 2004; Sundelin Wahlsten & Sarman, 2013). This is confirmed by our current results, with the rate of hyperactivity/inattention showing substantial mean differences between groups among the SDQ subscales at each assessment. Similar increases in conduct problems with child age particularly from early childhood, were also observed amongst opioid-exposed children. Collectively, these findings suggest that these children are likely to be at very high risk not just of more severe externalizing behavior problems in childhood, but also a poorer prognosis longer term. Finally, rates of peer (i.e., social) problems were also consistently elevated, and emotional problems of opioid-exposed children seemed to exacerbate over the course of childhood. Given the high rates of parental psychopathology and family instability in this high-risk group, a question for future study may be to examine whether early onset symptoms reflect attachment or social relationship related issues in early childhood. Further, assessments using standardized diagnostic measures of mental health disorders will also be important.

According to the Developmental Origins of Health and Disease (DOHaD) model, unfavorable conditions in utero (e.g., opioid exposure, chronic stress) affect behavioral functioning and health later in life via prenatal programing (Barker, 2007). This includes teratogenic effects on the developing central nervous system (Woodward et al., 2018), potentially increasing mental health vulnerability. DOHaD also proposes that long-term risk is initially induced through adaptive responses (e.g., changes in metabolism or tissue sensitivity to hormones) that the fetal organism makes to maternal cues about her health or physical state, leading to altered organ development. Two mechanisms related to prenatal programming may explain the behavior regulation difficulties seen in opioid-exposed children: The first suggests that opioids may alter the hypothalamic–pituitary–adrenal (HPA) axis (Sithisarn et al., 2008, 2017; Slamberova, Riley, & Vathy, 2005; Taylor, Soong, Wu, Yee, & Szeto, 1997), which has been linked, for instance, with ADHD (Ma, Chen, Chen, Liu, & Wang, 2011). In addition, prenatal and postnatal stressors may have additive or interactive programming influences on fetal HPA axis functioning, on top of prenatal opioid exposure (Lester & Padbury, 2009). For instance, opioid-using mothers may have a higher risk for depressive symptoms during and after pregnancy, and these could be associated with their caregiving quality, thereby directly and indirectly affecting infants’ stress regulation and behavior (Conradt et al., 2018). The second potential mechanism could be that prenatal opioid exposure alters the development of dopaminergic reward-related circuits, which may in turn lead to dysregulated, hyperactive behavior (Sithisarn et al., 2017). What’s more, DOHaD studies have documented a strong relationship between maternal depression and stress during pregnancy and children’s subsequent behavior regulation trajectories, even after controlling for confounders such as postnatal maternal depression (Henrichs & Van den Bergh, 2015). They also suggest that this relationship may be continuously modulated by environmental experiences (Henrichs & Van den Bergh, 2015). Our novel findings of the independent, additive contributions of prenatal risk, social risk, postnatal caregiving quality, and caregiver changes to predicting children’s emotional and behavioral problem trajectories add important information about the potential mechanisms placing infants born to opioid dependent mothers at increased psychopathological risk. For instance, the non-exposed children’s trajectories displayed in Fig. 4 show an expected small normative improvement in self-regulatory abilities from 2 to 4.5 years, but this pattern is less distinct among the opioid-exposed children. This suggests that some risk factors may be at play that dynamically alter the normative course of development over time. Importantly, our final growth curve model showed that the additive effects of prenatal and social risks remained significant even when effects of the postnatal rearing environment and primary caregiver changes were added to the model. Thus, the adverse developmental effects documented here cannot exclusively be attributed to the different postnatal environments that children grew up in, but prenatal and social mechanisms related to prenatal programming remain at play, even at later ages, over and above the effects of in-utero opioid exposure. These results offer critically important information for designing prevention and intervention services for current and future generations of opioid-using mothers and their offspring. Development is a complex multidirectional process that can be described as a constant feedback loop between biological and environmental factors, starting long before birth. Our findings suggest that intervention approaches that exclusively aim at modifying variables in children’s postnatal environments may not be able to effectively facilitate resilience for this at-risk group. Instead, integrated biopsychosocial approaches that start during or before pregnancy and target opioid-using women’s mental and physical health may be warranted in order to increase life chances for the next generation.

**Strengths and Limitations*.*** The findings presented here are from a prospective regional sample of opioid-exposed children and an equally-sized group of non-exposed age-matched controls. Compared to other longitudinal cohort studies, our sample is characterized by very high sample retention over time and age, a marker of excellent study quality. Alongside our opioid exposed group, measures were also collected on a randomly identified, regionally representative comparison group of non-exposed infants. This helped avoid problems associated with the Flynn effect and the use of normative cut-off points at very young ages, such as age 2. It also minimizes the likelihood that unintended and potentially unknown biases are introduced into the analysis. Assessments were rigorous and used internationally accepted, standardized measures at multiple times over the course of infancy and childhood. The rationale was hypotheses-driven and a sophisticated analysis approach helped uncover the complex mechanisms underlying developmental outcomes after in-utero opioid exposure.

However, the study also had some limitations. Our opioid-exposed sample only included infants of mothers who were in MMT treatment. Untreated opioid dependence is extremely rare in New Zealand, and these women were prioritized for substitution treatment which is free and accompanied by wrap-around psychiatric and obstetric care. Thus, results from these dyads may underestimate potential adverse effects of substance use on the child, and will have limited generalizability to infants of mothers who do not seek or receive substitution treatment when pregnant or when that care is unavailable.

One of our aims was to assess different pre- and postnatal biopsychosocial risks and their contributions to opioid-exposed children’s development. As in any study, our analyses were limited by the variables that had been assessed, and their respective distributions. For instance, a more process-oriented, longitudinal, and observation-based assessment of reciprocal caregiver-child interactions would help uncover the complex effects of social relationships on trajectories in future studies. Relatedly, many predictors and the dependent variables were based on caregivers’ reports, which can be biased (De Los Reyes & Kazdin, 2005; Van Roy, Groholt, Heyerdahl, & Clench-Aas, 2010). What’s more, we were not able to include information about other critically important domains that could explain mechanistic effects, such as (epi)genetics or stress response differences. The single stochastic imputation performed to maximize the utility of available data does not fully account for the variability structure of imputed and missing values, and hence may have introduced downward bias in the estimates of standard errors of model estimates (Carlin, Li, Greenwood, & Coffey, 2003; Rubin, 1987). In addition, statistical power to detect interaction effects between the different biopsychosocial risk scores was limited. Future studies with larger sample sizes will be important in helping to replicate our study findings and to further evaluate whether the effects of pre- and postnatal factors on child behavior are additive, interactive or both. A more in-depth examination of specific associations between some of the risk exposures we have identified and child outcomes may also be helpful, as this was beyond the scope of this integrated longitudinal analysis of the contributions of a wide range of potential pre- and postnatal explanatory factors.

**Conclusion*.*** Children who were exposed to opioids in-utero remain at risk for emotional and behavioral difficulties throughout the course of their childhood. In addition to the initial effects of opioids, a complex set of prenatal biological and postnatal environmental factors additively and dynamically contribute to substantial variations in children’s individual developmental trajectories. Future studies and evidence-based efforts to mitigate the long-term consequences of opioid use in pregnancy need to take children’s diverse biopsychosocial risks into account.

## Supplementary Information

Below is the link to the electronic supplementary material.

Supplementary file1 (PDF 128 KB)
