# Supplementary material for: Emotional and Behavioral Trajectories of 2 to 9 Years Old Children Born to Opioid-Dependent Mothers
Source: Res Child Adolesc Psychopathol. 2021 Jan 12;49(4):443–57. doi: 10.1007/s10802-020-00766-w (PMC7943531; doi:10.1007/s10802-020-00766-w)
Supplement: Supplementary file 1 — Supplementary file1 (PDF 128 KB) [file 10802_2020_766_MOESM1_ESM.pdf]

Supporting Information

Table S1
Measures of Maternal and Infant Characteristics

Measure	***Assessment Time(s) / Items***	***Coding***
Edinburgh Postnatal Depression Scale (EDPS)	Pregnancy, 18 months / 10 items assessing depressive symptomatology over the previous 2 weeks, each ranging from 0 to 3	Sum score, range 0-30
Smoking	Pregnancy / Self-reported average number of cigarettes per day in each trimester	Average number
Psychiatric illness	Pregnancy / Self-report of treatment sought for any mental illness	Binary (yes/no)
Maternal nutrition	Pregnancy / Total number of servings of fruit, vegetables, meat, bread, other cereals, milk, eggs consumed per week	Sum score, range 20-156
Illicit drug use	Birth – 4.5 years / Detailed self-reported drug use at each assessment point	Binary (yes/no)
Parenting Stress	18 months / 16-item scale assessing caregiver exposure to a range of stressors including insufficient financial resources, inadequate accommodation, no social support, and relationship difficulties	Sum score, range 16-36
Corporal punishment	18 months / Parent-Child Conflict Tactics Scale, 22 items assessing how parents disciplined their children using a range of methods including psychological aggression, non-violent discipline and physical assault. Parents completed the CTS-PC for every child in their care and were then assigned a physical punishment score based on the highest score obtained across all of their children.	Sum score, range 0-33
Home Observation for the Measurement of the Environment (HOME)	18 months / 6 scales incl. (1) emotional and verbal responsivity of the caregiver, (2) acceptance of suboptimal behaviour and avoidance of restriction and punishment, (3) organization of the physical and temporal environment, (4) provision of appropriate play materials, (5) parental involvement with the child, and (6) opportunities for variety in daily stimulation	Sum score, range 19-45
